# Roles of computational modelling in understanding p53 structure, biology, and its therapeutic targeting

**DOI:** 10.1093/jmcb/mjz009

**Published:** 2019-02-06

**Authors:** Yaw Sing Tan, Yasmina Mhoumadi, Chandra S Verma

**Affiliations:** 1 Bioinformatics Institute, Agency for Science, Technology and Research (A*STAR), 30 Biopolis Street, #07-01 Matrix, Singapore; 2 School of Biological Sciences, Nanyang Technological University, 60 Nanyang Drive, Singapore; 3 Department of Biological Sciences, National University of Singapore, 14 Science Drive 4, Singapore

**Keywords:** p53, structure, computational modelling, therapeutic targeting

## Abstract

The transcription factor p53 plays pivotal roles in numerous biological processes, including the suppression of tumours. The rich availability of biophysical data aimed at understanding its structure–function relationships since the 1990s has enabled the application of a variety of computational modelling techniques towards the establishment of mechanistic models. Together they have provided deep insights into the structure, mechanics, energetics, and dynamics of p53. In parallel, the observation that mutations in p53 or changes in its associated pathways characterize several human cancers has resulted in a race to develop therapeutic modulators of p53, some of which have entered clinical trials. This review describes how computational modelling has played key roles in understanding structural-dynamic aspects of p53, formulating hypotheses about domains that are beyond current experimental investigations, and the development of therapeutic molecules that target the p53 pathway.

## Introduction

Discovered in 1979, the tumour suppressor protein p53 is a transcription factor that regulates genes involved in cell cycle arrest, apoptosis, senescence, and DNA repair (Vogelstein et al., 2000; Toledo and Wahl, 2006). Over the years, it has become one of the most important and attractive drug targets in cancer therapy (Brown et al., 2009, 2011a). Its significance stems from its role as the ‘guardian of the genome’ (Lane, 1992), in which it coordinates cellular responses to various stress signals, including DNA damage, oxidative stress, heat shock, and oncogene activation. Impairment of p53 function by either mutation of the *TP53* gene or overexpression of negative regulators of p53 is a major cause of tumourigenesis.

**Figure 1:**
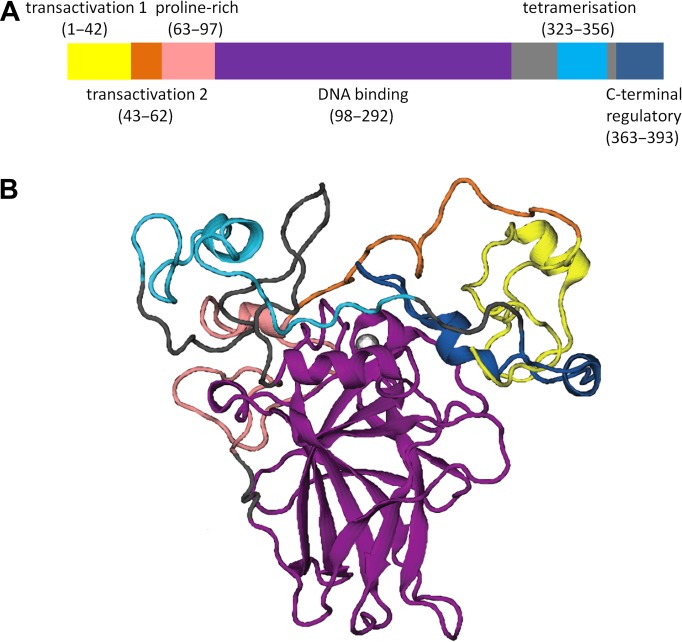
The p53 architecture. (**A**) Domain architecture of p53. (**B**) The modelled full-length structure of p53 (Chillemi et al., 2013). Reprinted by permission of Taylor & Francis Ltd.

p53 functions as a tetramer. Each monomer consists of an intrinsically disordered N-terminal transactivation domain (TAD), a proline-rich domain, a core DNA-binding domain (DBD), a tetramerization domain, and a C-terminal regulatory domain (CTD) (Figure 1). The first experimental structures of p53 were solved in 1994. One of them reveals how the DBD is bound to DNA (Cho et al., 1994), while the other shows how the p53 tetramer is formed from the assembly of a dimer of dimers of the tetramerization domain (Clore et al., 1994). The crystal structure of a peptide derived from the p53 TAD in complex with one of its negative regulators, MDM2, was obtained two years later in 1996 (Kussie et al., 1996). The number of p53-related structures deposited in the Protein Data Bank (PDB) has proliferated exponentially since then, providing a rich resource for computational modelling.

The plethora of experimental structures has made it possible for computational modellers to further build upon our knowledge of p53. A variety of computational approaches, including homology modelling, docking, molecular dynamics (MD), have been employed to study the domain structure and dynamics of both wild-type and mutant p53. Not only does p53 interact with DNA, it is also a hub protein that is central to the normal function and stability of the protein–protein interaction (PPI) network in an organism (Collavin et al., 2010). A search of public PPI databases using the APID web server (Alonso-López et al., 2016) showed that human p53 is involved in >1100 PPIs. Computational modelling approaches complement structural biology approaches in understanding these interactions at the atomic level. MD simulation methods provide an additional temporal perspective.

Therapeutic targeting of p53 focuses on the discovery of molecules that either inhibit its negative regulators or stabilize its mutants. Computational methods have not only provided insight into the structure and dynamics of p53, but also played important roles in the discovery of many of these therapeutic molecules (Lauria et al., 2010). They help to provide insight into the mechanism and energetics of binding, and effect of ligand binding on the dynamics and structure of p53 and its binding partners. In many cases, the discovery of the lead compound was driven by computational molecular models, thus reducing the need for tedious and expensive screening of extensive compound libraries.

In this review, we summarize and discuss the contributions that computational modelling has made towards our understanding of p53 structure, biology, and its therapeutic targeting over the last 20 years.

## Understanding p53 structure

### Wild-type p53

Experiments have shown that the TAD adopts transiently stable secondary structures. MD simulations of the TAD agree with the experimental observations and provide further information about its structure and dynamics. They show that the TAD exists in a partially collapsed state (Lowry et al., 2007), including the region from Phe19 to Leu22, which exhibits local helix propensity (Espinoza-Fonseca and Trujillo-Ferrara, 2006), and that leucine-rich clusters are responsible for stabilizing its folded state (Espinoza-Fonseca, 2009).

The DBD contains an antiparallel β-sheet sandwich framework held together by weakly conserved loops. Loops L2 and L3 accommodate a tetrahedrally coordinated Zn^2+^ ion. Although the role of zinc in maintaining the stability of p53 was known, mechanistic details were lacking. MD simulations of the DBD with and without Zn^2+^ were carried out to study its role in DNA recognition and DBD stability (Duan and Nilsson, 2006). The inherent instability of p53 DBD was also investigated by Verma and coworkers (Madhumalar et al., 2008) in MD simulations, who were inspired to explain why a double mutation of p53 to the corresponding residues in the comparatively stable homologues p63 and p73 stabilizes the DBD, as reported in an earlier work by Fersht and coworkers (Cañadillas et al., 2006). Other computational studies involving the use of MD simulations and homology modelling have been performed to understand the molecular basis for the low thermal stability of human p53 DBD compared to its homologues, p63 and p73 (Patel et al., 2008a), and orthologues from evolutionarily less developed organisms (Pan et al., 2006; Pagano et al., 2013). Further insights into the dynamics of the highly flexible loop L1 were gained in multiple MD simulations of the DBD (Lukman et al., 2013).

The oligomerization of p53 is mediated by the tetramerization domain. Its structure was resolved as early as 1994 (Clore et al., 1994; Lee et al., 1994), but it was only in 2005 that the first MD simulations of this domain were performed to examine how the domain folds and oligomerises (Chong et al., 2005; Duan and Nilsson, 2005).

Following the success of these early simulations in providing important insights into the structure and dynamics of individual p53 domains, several groups became interested in studying the full-length p53 protein in silico. In 2011, Khazanov and Levy (2011) carried out coarse-grained simulations of the full-length p53 tetramer to study its movement along DNA. The first atomistic MD simulations of the full-length p53 monomer and tetramer in complex with DNA were reported by Chillemi et al. (2013) and D’abramo et al. (2016), respectively. A subsequent simulation study of the p53 tetramer–DNA complex suggests for the first time that the CTD directly interacts with DNA (Demir et al., 2017).

### Mutant p53

The molecular modelling and simulation of mutant forms of p53 are of exceptional interest because of their association with cancer development. Although the majority of p53 cancer mutations occur in the DBD, they can also be found in other domains, including the TAD and tetramerization domain. Conformational ensembles of TAD mutants have been studied using replica exchange implicit solvent MD simulations, which show that the mutations cause local and remote structural changes that may affect binding to p53 partner proteins (Ganguly and Chen, 2015). The pH-dependent destabilizing effect of the R337H mutation on the tetramerization domain was studied using MD simulations by Bashford and coworkers (Lwin et al., 2007). They found that Arg337 constitutes part of a salt bridge cluster that stabilizes the wild-type tetramer. Under alkaline conditions, the R337H mutant is destabilized by the deprotonation of His337, which loses interaction with the salt bridge cluster.

One of the earliest computational studies of p53 was carried out by Brandt-Rauf’s group, who studied the structures of p53 DBD peptide mutants using a conformational analysis program (Brandt-Rauf et al., 1992, 1994). His group was also the pioneer in p53 MD simulations. The release of the first crystal structure of the p53 DBD in 1994 made it possible to study the entire domain computationally. Brandt-Rauf and his colleagues interrogated the associated dynamics of this structure and two mutants in short MD simulations, which show that the PAb240 epitope is exposed in the mutants only. This was subsequently validated using immunohistochemistry of liver angiosarcomas. The success of this early work established MD simulation as a method with good predictive power, which was later extended to the study of the H179L (Chen et al., 1999), L145Q, V157F, R282W (Calhoun and Daggett, 2011), and environmentally-induced mutants (JM Chen et al., 2001). Later simulation studies that incorporate DNA allowed the mechanism of mutation-induced loss of p53–DNA contacts to be elucidated (Lu et al., 2007). A few oncogenic DBD mutants are temperature-sensitive. They bind to DNA at lower temperatures but lose binding at warmer temperatures. MD simulations have been used to understand the mechanism for their temperature-induced oncogenicity, which involves solvent exposure of interface residues and changes to loop dynamics (Barakat et al., 2011; Koulgi et al., 2015; Ng et al., 2015).

Not all mutations in the DBD are cancer-causing. Some of them are benign. MD simulations were coupled with a free energy method called molecular mechanics/Poisson-Boltzmann surface area (MM/PBSA) to explain why the C238Y mutant retains wild-type activity (Ferrone et al., 2006). More intriguingly, some DBD mutations actually suppress the effect of oncogenic mutations. The molecular mechanisms of these so-called second-site suppressor mutations were first studied computationally by Lim and coworkers (Wright et al., 2002). MD simulations of wild-type p53, the R273H mutant and the R273H+T284R mutant in their unbound and DNA-bound states were performed to understand the detrimental effect of R273H and suppressing effect of T284R. The simulations show that loss in DNA binding of the R273H mutant results from the disruption of a hydrogen bond network, which in turn disrupts major groove interactions of R280 and K120. The positively charged Arg284 forms a new interaction with DNA that helps to restore the DNA contacts of R280 and K120. Other second-site suppressor mutations that have been studied by MD simulations include N235K (Tan et al., 2009) and H115N (Merabet et al., 2010). An MD study of several DBD mutants found that in general, oncogenic mutations increase flexibility while second-site suppressor mutations reduce flexibility (Demir et al., 2011).

## Understanding p53 interactions

### Interaction with DNA

The interaction between p53 DBD and DNA has been studied at the atomic level in several MD simulation studies. These simulations sought to elucidate the mechanism of DNA recognition and binding, the structural implication of DBD binding to DNA, and the effect of DNA binding on cofactor recruitment by the DBD (Pan and Nussinov, 2010a; Lambrughi et al., 2016). MD simulations of the p53 tetramer–DNA complex suggest that only two monomers are involved in DNA recognition and that at least three R273H mutant monomers are required to disable tetramer binding to DNA (Ma and Levine, 2007). MD simulations performed by Pan and Nussinov have also shown that DNA bends in response to DBD binding and the amount of bending is sequence-specific, in agreement with experimental data (Pan and Nussinov, 2007). The remarkable agreement with experimentally observed differential bending patterns suggests that the models proposed in this study are functionally relevant. The role of DNA sequence and flexibility in p53-induced DNA bending was uncovered in a later study by the same authors (Pan and Nussinov, 2008). They further investigated the relationship between organization of the p53 response elements and binding cooperativity. MD simulations of the tetrameric p53–DNA complex followed by conformational analysis show that p53–DNA binding is highly cooperative when there are two or fewer base pair spacers between the p53 response elements (Pan and Nussinov, 2009). To understand the binding process, Pan and Nussinov (2010b) have also performed MD simulations of p53 DBD approaching DNA. They found that initial electrostatic recognition between the positively charged DNA binding surface of DBD and the negatively charged DNA results in the drifting of the DBD along the DNA surface until the key binding residues anchor into the major groove.

### Protein–protein interactions

The different domains of p53 are involved in a myriad of interactions with various proteins. Some of these partner proteins engage with more than one p53 domain. Molecular models and MD simulations can help us to understand the structure and dynamics of these protein–protein complexes, and the effects of post-translational modifications on their interactions.

The binding of p53 TAD to MDM2 was studied in great detail by Chen and Luo, who performed MD simulations on apo MDM2, apo p53 and the MDM2–p53 complex for a total of 600 ns (Chen and Luo, 2007). The simulations show that the p53 TAD binding pocket of MDM2 is, as expected, narrow in the apo state and expands upon binding p53. Later in 2012, Verma and coworkers (Dastidar et al., 2012) performed MD simulations of the p53 TAD approaching MDM2. These simulations postulate the initial capture of Phe19, which acts as an anchor that opens up the binding cleft through crack propagation. This could explain why the F19A p53 mutant does not bind to MDM2 (Bottger et al., 1997). The detrimental effect of the phosphorylation of p53 Thr18 (Lee et al., 2007), p53 Ser20 (ElSawy et al., 2015), MDM2 Ser17 (Dastidar et al., 2011), and MDMX Tyr99 (Chan et al., 2017) on p53 binding to MDM2 or MDMX have also been extensively studied in MD and Brownian dynamics (BD) simulations.

Nussinov and coworkers (Tuncbag et al., 2009) provided a temporal dimension to the PPI network of the p53 hub protein based on its DBD interactions. This allows for the prediction of p53 interactions that can and cannot occur at the same time. MD simulations and protein–protein docking have been used to study the interaction of the DBD with the ASPP proteins (Patel et al., 2008b), Hsp90 (Blacklock and Verkhivker, 2013), and ubiquitin (Landré et al., 2017). The computational model of the DBD–ubiquitin complex is especially illuminating as it shows that ubiquitin can extend the DNA binding interface of the DBD, thus explaining why monoubiquitination, which occurs under certain cellular conditions such as post-nutlin treatment, counter-intuitively enhances p53 activity.

The CTD of p53 is intrinsically disordered, allowing it to adopt multiple conformations for binding to different protein partners. Its interaction with S100B was particularly well characterized in several computational studies that employed simulation methods such as MD and Monte Carlo (Chen, 2009; Staneva et al., 2012; McDowell et al., 2013). MD simulations have also been combined with free energy techniques to examine the effect of K382 acetylation on p53 binding to the CBP bromodomain (Eichenbaum et al., 2010). A couple of comprehensive MD simulation studies on the different CTD complexes helped to provide important insights into the ability of the CTD to engage with diverse interfaces (Allen et al., 2010; Kannan et al., 2016).

## Therapeutic targeting of p53

Impairment of p53 function is caused by either mutations to the *TP53* gene or overexpression of proteins that negatively regulate p53 activity, such as MDM2 and MDMX. It is estimated that mutations in *TP53* are implicated in about half of all human cancers (Vousden and Lu, 2002). Most of these mutations (95%) occur in the DNA binding domain, which could undermine the direct binding of p53 to DNA, destabilize the protein so that it unfolds, or induce misfolding (Vousden and Lu, 2002). Therapeutic intervention seeks to restore proper p53 function either by reactivating mutant p53 or enhancing the activity of wild-type p53 by inhibition of its interaction with MDM2 and/or MDMX. This section highlights the important roles that computation has played in the development and discovery of drugs that target the p53 pathway.

### Targeting the p53–MDM2 interaction

#### Small molecules

p53 interacts with the N-terminal domains of MDM2 and MDMX via its N-terminal TAD. The crystal structure of MDM2 complexed with a short peptide corresponding to the N-terminal region of p53 shows that the interaction is mediated by three critical residues from p53: Phe19, Trp23, and Leu26 (Kussie et al., 1996). These residues insert into a deep hydrophobic cleft in MDM2. Early computational efforts focused on the generation of pharmacophore and quantitative structure–activity relationship (QSAR) models based on this protein–peptide complex structure. In 2001, Galatin and Abraham (2001) reported the development of a QSAR model that predicts peptide activity based on hydropathic descriptors obtained from the hydropathic interactions (HINT) program. HINT was also used to generate a p53 pharmacophore model by analysis of importance of individual atoms within the side chains of Phe19, Trp23, and Leu26. Based on this model, a 3D database search of the National Cancer Institute (NCI) chemical database was carried out using the program UNITY (Galatin and Abraham, 2004). This yielded a sulphonamide compound that exhibits low micromolar inhibition of the p53–MDM2 interaction in binding assays and increases p53-dependent transcription in cancer cells. Zhao et al. (2002) employed a similar pharmacophoric search with UNITY and evaluated the hit compounds by docking. One of them was found to activate the p53 pathway in several tumour cell lines.

Molecular docking played a prominent role in the development of later generations of inhibitors. The first isoindolinone-based inhibitors of the p53–MDM2 interaction identified in a preliminary binding assay had very weak activity (Hardcastle et al., 2005). They were optimized with the help of the structure-based *de novo* ligand design program SkelGen (Dean et al., 2006) and docking programs, easyDock (Mancera et al., 2004) and GOLD (Jones et al., 1997). A new series of inhibitors based on the isoindolinone scaffold, the most potent of which has low micromolar IC50 in an ELISA assay, was generated (Hardcastle et al., 2006). Further optimization of this series of inhibitors, guided by NMR titrations, resulted in compounds with nanomolar potency and improved cellular activity (Hardcastle et al., 2011).

The structure-based design of another series of MDM2 inhibitors, the spiro-oxindoles, was also guided by molecular docking. Candidate compounds containing the spiro-oxindole scaffold were docked into MDM2 using GOLD, yielding a lead compound that had slightly weaker binding affinity for MDM2 than the p53 peptide (Ding et al., 2005). Based on its predicted binding mode obtained by docking, modifications to the compound structure were made to enhance hydrophobic interactions. The modified compound is ~100 times more potent than the initial lead compound. It was further optimized to improve its binding and pharmacokinetic properties, which eventually led to the identification of MI-219, a potent, highly specific and orally active inhibitor that has entered clinical trials (Ding et al., 2006; Shangary et al., 2008).

Although virtual screening by pharmacophore search is fast, it cannot predict quantitatively how tightly the ligand binds to the target protein. Conversely, although virtual screening by docking allows for quantitative assessment of the binding affinity of a compound for the target protein, it is relatively computationally expensive. An integrated approach that combines these complimentary computational techniques was adopted by some groups to discover new MDM2 inhibitors. The same group that developed MI-219 separately discovered a nanomolar inhibitor of the p53–MDM2 interaction using an integrated virtual screening approach (Lu et al., 2006b). They initially did a pharmacophore search of NCI’s database, which led to the identification of 2599 hits. These hit compounds were then docked into the p53 binding site of MDM2. Some of the top-ranking compounds were evaluated in binding and cellular assays, resulting in the discovery of a potent quinolinol inhibitor. Another research group used the program CAVEAT (Lauri and Bartlett, 1994) to search chemical databases for scaffolds similar to that of the p53 peptide (Lu et al., 2006a). These hit scaffolds were docked to MDM2 using DOCK (Ewing et al., 2001), followed by clustering of promising scaffolds and filtering away of ‘poor’ scaffolds. A final round of docking was performed with the side chains added to the scaffolds. This yielded a library of moderately active MDM2 inhibitors with α-helix-mimetic scaffolds.

To expand the scaffold diversity of lead MDM2 inhibitors, Czarna et al. (2010) employed multicomponent reaction chemistry (MCR) complimented by docking. They first created a virtual library of compounds containing the indole moiety of Trp23, which was identified as the anchor residue. These compounds were docked into the p53 binding site of MDM2 using the software suite Moloc (Gerber and Müller, 1995). The highest-ranked compounds were then selected for synthesis by MCR. Seven scaffolds exhibited low micromolar activity in NMR-based binding assays.

Initial pharmacophore and docking models were based on the single static crystal structure of MDM2 bound to the p53 peptide. The importance of protein flexibility for structure-based drug design was later recognized, as research groups began to incorporate it into their computational models. Bowman et al. (2007) proposed the use of the multiple protein structure (MPS) method to generate a dynamic receptor-based pharmacophore model of the p53 binding site in MDM2. Protein structures were extracted at regular intervals from an MD simulation of p53-bound MDM2. These structures were flooded with benzene, ethane and methanol probes to identify consensus interaction sites within the p53 binding cleft. A database of ~35000 commercially available compounds was then screened against this receptor-based pharmacophore model. Five active compounds were eventually identified. The binding modes of these inhibitors were investigated by induced-fit docking (Sherman et al., 2006), which accounts for protein flexibility by allowing for limited movement of residues close to the ligand during the docking.

The p53–MDM2 interaction was proving to be a highly tractable drug target and pharmaceutical companies eventually got into the act of designing inhibitors against this interaction. Computational modelling played important roles in the development of some of these drug candidates. For example, Amgen initiated their rational design of MDM2 inhibitors by using both ligand-based and structure-based computational techniques to identify suitable scaffolds (de Turiso et al., 2013). This led to the discovery of AM-8553 (Rew et al., 2012) and ultimately AMG 232 (Sun et al., 2014), which is currently undergoing clinical trials for cancer treatment. Norvatis also made concerted efforts to discover small-molecule inhibitors of the p53–MDM2 interaction, which led to the identification of three different classes of inhibitors. The first class of inhibitors has 3-imidazolyl indole as the core structure (Furet et al., 2012), which was designed with the help of molecular modelling and docking by the program MacroModel (Mohamadi et al., 1990). The structure-based optimization of the second class of inhibitors, the tetra-substituted imidazoles, was guided by molecular models obtained from docking (Vaupel et al., 2014). The third class of inhibitors, the dihydroisoquinolinones, was discovered by virtual screening of ~50000 compounds from the Novartis compound collection (Gessier et al., 2015). This integrated virtual screening approach combines the methods of QSAR, high-throughput docking, and pharmacophore modelling (Jacoby et al., 2009). The initial hit compound identified was optimized into NVP-CGM097, which is currently under evaluation in clinical trials (Holzer et al., 2015).

**Figure 2:**
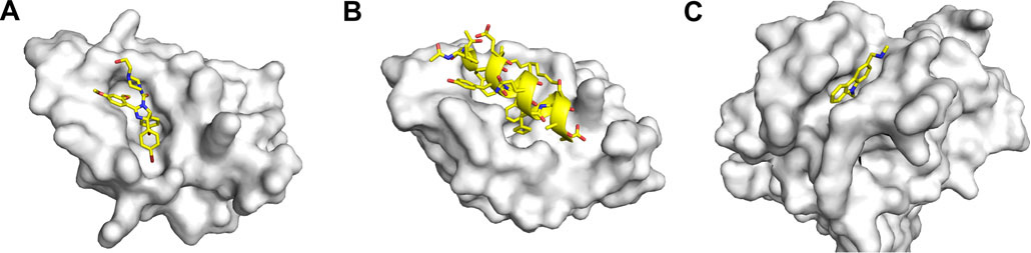
Structures of molecules developed to modulate the p53 pathway. (**A**) Nutlin-2 in complex with MDM2 (PDB 1RV1) (Vassilev et al., 2004). (**B**) ATSP-7041 in complex with MDMX (PDB 4N5T) (Chang et al., 2013). (**C**) PhiKan083 in complex with p53 DBD (PDB 2VUK) (Boeckler et al., 2008).

Besides virtual screening and structure-based design, computational methods have also been used to elucidate the mechanisms by which these small molecules bind to MDM2. The nutlins are the first reported class of small-molecule p53–MDM2 inhibitors with in vivo activity (Figure 2A) (Vassilev et al., 2004). However, they are unable to disrupt the p53–MDMX interaction. MD simulations of their complexes with MDM2 and MDMX were performed to understand the molecular basis for their binding specificity (Joseph et al., 2010b). The reduced interaction of nutlin with MDMX compared to MDM2 is attributed to the replacement of Leu54 in MDM2 with the larger Met53 in MDMX. This reduces the size of the binding cleft and hence, the interactions of nutlin with MDMX. The mechanism of nutlin binding to MDM2 was also investigated using BD and MD simulations in a few separate studies. The most potent nutlin variant is nutlin-3, which has two chiral centres. BD simulations of the association of the four different nutlin-3 stereoisomers with MDM2 was performed to explain the stereoselectivity of MDM2 for one of the stereoisomers (ElSawy et al., 2013). In another study, a putative second nutlin interaction site was discovered on MDM2 by hydrogen/deuterium exchange mass spectrometry (Hernychova et al., 2013). MD simulations of the unbound nutlin and MDM2 suggest that this region could serve as an initial docking site for nutlin, which then enters the p53-binding pocket after a series of dissociations and reassociations from the MDM2 surface.

Besides nutlin, other MDM2 inhibitors have also been studied using computational methods. During the optimization of their lead compound to AM-8553, Rew et al. (2012) did quantum mechanical calculations on some of the derivative compounds to predict the relative stability of their anti and gauche conformations. It was found that potency was increased if the more stable conformer of the free compound matches its bound conformation within MDM2, which helped to rationalize the experimental potency data. A comprehensive computational study that involved enhanced sampling techniques, umbrella sampling and variational free energy profile methods helped to shed light on the effect of ligand binding on the dynamics and structure of the N-terminal lid region of MDM2 (Bueren-Calabuig and Michel, 2015). Their results indicate that the binding of nutlin and benzodiazepinedione-based MDM2 inhibitors are unaffected by the lid, while piperidinone-based MDM2 inhibitors gain potency by interaction with the lid. The piperidinones form stabilizing hydrophobic contacts with residues at the base of the lid that induce the folding of MDM2 residues 19–24 into an α-helical conformation. These findings provide suggestions on how small-molecule MDM2 inhibitors could be designed for optimal interaction with the lid.

#### Peptides

Peptides are promising therapeutic agents for the modulation of PPIs (Wilson, 2009). They can bind with high specificity and potency to their target proteins, resulting in reduced off-target effects and improved safety (Craik et al., 2013). However, peptides are hindered by their poor pharmacokinetic properties, such as low bioavailability, rapid elimination, poor in vivo stability, and the requirement for intravenous administration. Computational tools have mainly been used to understand the energetics and mechanism of p53 peptide binding to MDM2, and to a limited extent, to design novel peptidic inhibitors of the p53–MDM2 interaction.

The p53–MDM2 interaction was chosen as the model system by Massova and Kollman (1999) to validate the computational alanine scanning technique. In their study, the computed change in binding free energy upon alanine mutation of the p53 peptide residues agreed qualitatively with the experimental data. The three key residues, Phe19, Trp23, and Leu26, as well as Leu22, were found to be the most important residues for p53 binding to MDM2. Structures used for the calculation were taken from a 400 ps MD simulation of the p53–MDM2 complex. In 2005, Zhong and Carlson (2005) performed computational alanine scanning on the same system. The differences were that the structures were derived from a longer 2 ns MD simulation, selected MDM2 residues were mutated, and the faster molecular mechanics/Generalized Born surface area (MM/GBSA) (Srinivasan et al., 1998) method was used to calculate the energies instead of the molecular mechanics/Poisson Boltzmann surface area (MM/PBSA) (Kollman et al., 2000) method. The information was used to design a p53 peptidomimetic with a β-proline scaffold. Although the β-peptide mimic was not tested experimentally, it was predicted to have a lower free energy of binding than the p53 peptide based on MM/GBSA calculations.

Using a similar protocol of MD simulations followed by free energy calculations, Madhumalar et al. (2009) designed a peptide that was predicted to have higher affinity for MDM2 than the p53 and 12-1 peptides, the latter of which is a high-affinity MDM2-binding peptide derived from phage display experiments. This study showed that certain residues in the p53 peptide could be mutated to enhance its binding affinity for MDM2. Pro27 is of particular interest, as it has been shown that its mutation to Ser improves the binding of the p53 peptide to MDM2 considerably (Zondlo et al., 2006). MD simulations revealed that both helical and extended C-terminal conformations of the P27S mutant peptide have similar binding affinities because of enthalpy-entropy compensation (Dastidar et al., 2008). A later study attempted the mutation of C-terminal Pro27 in a truncated form of the p53 peptide to Ser, Thr, Ala, and Asn, all of which improved the binding affinity (Brown et al., 2011b). The kinetics of the binding of these peptides to MDM2 was subsequently studied using BD (ElSawy et al., 2016). It was found that there is a direct correlation between the peptide’s residence time around MDM2 and its binding affinity, with the tightest-binding P27S mutant peptide having the longest residence time.

#### Stapled peptides

The emergence of the peptide stapling technique in the last two decades has prompted a radical change in mindset regarding the use of peptide-based drugs. Peptide stapling essentially involves the covalent linkage of two appropriately-spaced and functionalised residues to form a cyclic peptide. The most popular stapling technique is hydrocarbon stapling, in which two olefin-bearing residues are crosslinked by a ruthenium-catalyzed ring-closing metathesis reaction to form an all-hydrocarbon staple (Schafmeister et al., 2000). Hydrocarbon-stapled peptides have been shown to exhibit enhanced α-helicity, potency, protease resistance, and cell permeability compared to their linear counterparts (Verdine and Hilinski, 2012), making them highly promising candidates for the therapeutic inhibition of PPIs. The p53–MDM2 interaction was one of the first to be targeted by stapled peptides, the most successful of which has reached clinical trials (Figure 2B) (Chang et al., 2013). Computational methods have played prominent roles in the elucidation of the mechanism of stapled peptide binding to MDM2 and the design of new stapled peptide inhibitors of MDM2 (Tan et al., 2016a).

The first stapled peptide inhibitors of MDM2 were reported by Bernal et al. in 2007. A hydrocarbon staple was installed on a series of p53-based peptides at the *i*-th and *i* + 7-th positions to improve their α-helicities. The most drug-like stapled peptide, SAH-p53-8, exhibits improved α-helicity, MDM2-binding affinity, and cell penetration over the wild-type p53 peptide. MD simulations of the stapled peptide complexes suggest that the hydrocarbon staple of SAH-p53-8 actually lies very close to the MDM2 surface, such that they form extensive hydrophobic contacts with each other, which further improve the binding affinity of the stapled peptide compared to the linear p53 peptide (Joseph et al., 2010a). The computational model was subsequently validated by an X-ray crystal structure of the SAH-p53-8–MDM2 complex (Baek et al., 2012), which revealed the intimate contacts between the hydrocarbon staple and the MDM2 surface. A subsequent study used multiple MD simulations of peptides displaced from the p53 binding pocket to understand the effect of hydrocarbon stapling on peptide binding to MDM2 (Sim et al., 2014). The wild-type p53 peptide was found to preferentially rebind by tilting towards the Phe19 binding site, while the stapled peptide has equal chance of tilting towards the Phe19 or Leu26 binding site during the rebinding process. This is attributed to the rigidity introduced by the staple and the absence of an extended C-terminal region. The simulations also show the formation of an ordered water network between the staple and protein surface as the stapled peptide rebinds to MDM2. An ensuing MD simulation study restrained the displaced peptides at their initial positions instead of allowing rebinding (Sim and Verma, 2015). These simulations suggest that the hydrogen-bonded interfacial water molecules facilitate binding by lowering the energy penalty associated with dehydration of the peptide–protein interface, which could have implications on the binding kinetics of the stapled peptide.

The potential of MD simulations to inform the rational design of stapled peptide inhibitors of MDM2 was highlighted by Tan et al. in their work on ligand-mapping molecular dynamics (LMMD). First implemented in 2012, LMMD involves the use of benzene molecules in explicit-solvent MD simulations to probe for hydrophobic binding sites on proteins (Tan et al., 2012). The method was later validated on several proteins with hydrophobic peptide binding sites and hydrocarbon staple interaction sites, including MDM2 (Tan et al., 2015). The simulations also yielded novel binding sites that have not been exploited for ligand binding. Indeed, two such putative binding sites near the p53-binding cleft were identified. Hydrocarbon-stapled peptides were rationally designed to target one of them (Tan et al., 2016b). However, X-ray crystal structures of the complex show the peptides interacting with the other putative site instead. This is because of the unexpected folding of the peptides’ C-termini into an α-helix. Nevertheless, this work has led to the development of a new series of MDM2-binding stapled peptides with improved binding affinities compared to the parent stapled peptide, and provides a proof of concept for the use of LMMD in the prediction of novel binding sites.

### Reactivation of mutant p53

The majority of mutations in the DBD of p53 are missense mutations. These point mutations can be classified as either contact or structural mutations. Contact mutations occur at the DNA binding interface and disrupt the binding of the mutant p53 to DNA. Otherwise, the overall structure of the contact mutant is similar to that of the wild-type protein. Conversely, structural mutations prevent proper folding of the domain, which causes conformational changes that either destabilize the mutant or prevent it from binding to DNA. Destabilized structural mutants may be able to bind small molecules that stabilize the functional conformation of the domain, thus restoring p53 activity. These molecules can target either several mutants or a specific mutant only. Computational tools have proven instrumental in the identification of druggable pockets within the mutant proteins and the discovery of ligands that bind to these pockets.

#### Rescuing the Y220C mutant

The Y220C mutation is the ninth most frequent p53-associated cancer mutation and the most common core domain structural mutation (Olivier et al., 2002), accounting for ~75000 new cancer cases each year (Petitjean et al., 2007). The mutation creates an exposed cleft that destabilizes the DNA binding domain (Joerger et al., 2006). This pocket was found to be druggable after a virtual screening of >2.5 million compounds from the ZINC database followed by NMR screening yielded a micromolar (150 μM) carbazole-based lead compound, PhiKan083 (Boeckler et al., 2008). The stabilizing effect of PhiKan083 was demonstrated in thermal stability experiments, which showed that PhiKan083 raises the melting temperature and slows the denaturation rate of the Y220C mutant. A new lead compound was subsequently discovered by screening a library of halogen-enriched small molecules (Wilcken et al., 2012). Lead optimization was guided by docking and involved the extension of the lead compound into two subsites within the Y220C binding pocket. This eventually led to the discovery of a low micromolar (9.7 μM) hit compound, PhiKan5196, which induced apoptosis in Y220C-containing human cancer cells.

The crystal structure of PhiKan083 in complex with the Y220C mutant reveals small conformational changes of the Y220C cavity upon ligand binding (Figure 2C) (Boeckler et al., 2008). To explore the structural plasticity of the cavity and identify binding subsites within the cavity, MD simulations of the Y220C mutant in a solution of water and 20% (*v/v*) isopropanol were performed (Basse et al., 2010). This method is related to LMMD and was first described by Seco et al. (2009). However, unlike LMMD, isopropanol probes were used to identify both polar and nonpolar binding sites. The simulations show that the cavity is highly flexible and fluctuates significantly in size. MD simulations of the Y220C mutant complexed with some of the fragment hits identified from fragment-based screening show that the fragments significantly reduce the flexibility of proline-rich loops lining the pocket, thus stabilizing the local protein structure.

Analysis of the crystal structures of the Y220C mutant bound to these fragment hits indicates the presence of a cryptic subpocket within the Y220C cavity. This subpocket is modulated by the Cys220 side chain and utilized by some of the fragments for binding. To obtain insights into this cryptic subpocket, long MD simulations (300 ns) of the apo mutant protein were performed (Joerger et al., 2015). The open and closed states of the subpocket were populated with comparable frequency in the simulations, which suggests that it is highly accessible for ligand binding.

Quantum mechanical calculations have been used to rationalize the reduced affinity caused by bioisosteric replacement in one of the p53-Y220C small-molecule stabilisers, PK5176 (Wilcken et al., 2015). This compound has an aromatic iodine atom that forms a halogen bond with a backbone oxygen within the Y220C cavity. Substitution of this atom with an ethynyl moiety led to a 13-fold loss in binding affinity. The ab initio calculations show that the complex formation energy for the ethynyl analogue is unfavourable. This is likely because of a combination of iodine’s superior polarisability, its more suitable interaction geometry, and the better fitting of the iodo analogue within the binding pocket.

#### Mutant stabilization via cysteine alkylation

Besides targeting a mutation-induced cavity, mutant p53 can also be stabilized by alkylation of solvent-exposed cysteines. Ligands that form covalent adducts with p53 and increase the thermal stability of several p53 mutants have been identified by fragment screening (Kaar et al., 2010). These alkylating agents were shown to react with several cysteines, of which Cys124 and Cys141 were found to be the most reactive by mass spectrometry experiments. However, crystal structures of the human p53 DBD show that the sulphydryl group of Cys124 is partially buried and not readily accessible to small molecules (Cho et al., 1994; Wang et al., 2007). MD simulations of wild-type p53 and three frequent cancer mutants reveal the formation of a transient pocket bounded by loop L1 and sheet S3, which exposes the side chain of Cys124 to the solvent (Wassman et al., 2013). Alkylating compounds known to cause p53 reactivation were docked into the open L1/S3 pocket of representative structures taken from the simulations. All of them were able to attain low-energy docked poses with their reactive methylene group in close proximity to the side chain of Cys124, thus supporting the existence of a druggable pocket in the L1/S3 region. To identify putative ligands of this pocket, an ensemble-based virtual screen based on the relaxed complex scheme (Lin et al., 2002) was carried out. These compounds were docked into representative structures taken from the MD trajectory of the R273H cancer mutant. Out of the 45 compounds selected for biological assay, stictic acid was the only one that reactivated mutant p53 in human cancer cells.

## Conclusion

As the guardian of the genome, p53 plays a vital role in suppressing tumour formation. The elucidation of its first structures in the mid-1990s kick-started intense efforts to develop p53-reactivating drugs for anticancer treatment. These efforts were aided by the use of computational modelling methods that leveraged the growing database of high-resolution structures of p53 itself and its complexes to provide valuable insights into their structure, mechanics, energetics, and dynamics. Notably, the discovery of several therapeutic molecules that restore p53 function, some of which are in clinical trials, were driven by computational models.

The rise of machine learning in recent years portends a change in the way we study p53 in our computers. Machine learning methods are expected to play increasingly prominent roles in drug discovery, especially with the emergence of deep learning as a game-changer in many fields of science (Ekins, 2016; Gawehn et al., 2016; Chen et al., 2018). They have proven beneficial in the early stages of drug discovery, particularly in the development of QSAR models and prediction of absorption, distribution, metabolism, excretion, and toxicity (ADMET) properties (Panteleev et al., 2018). They have also been applied to the development of scoring functions for docking (Ragoza et al., 2017) and *de novo* design (Olivecrona et al., 2017). Although the application of machine learning methods to the study of p53 has been limited so far (Danziger et al., 2007; Ramani and Jacob, 2013a, b), we anticipate that they will play significant roles in the development of new therapeutics to target p53 in the near future. Together with the more traditional computational modelling methods outlined in this review, they will allow us to unlock even more secrets of the guardian of the genome.
